# Unveiling mutational dynamics in non‐small cell lung cancer patients by quantitative *EGFR* profiling in vesicular RNA

**DOI:** 10.1002/1878-0261.12976

**Published:** 2021-05-20

**Authors:** Luigi Pasini, Michela Notarangelo, Alessandro Vagheggini, Marco Angelo Burgio, Lucio Crinò, Elisa Chiadini, Andrea Iamurri Prochowski, Angelo Delmonte, Paola Ulivi, Vito Giuseppe D'Agostino

**Affiliations:** ^1^ Bioscience Laboratory IRCCS Istituto Romagnolo per lo Studio dei Tumori (IRST) “Dino Amadori” Meldola Italy; ^2^ Laboratory of Biotechnology and Nanomedicine Department of Cellular, Computational and Integrative Biology (CIBIO) University of Trento Italy; ^3^ Unit of Biostatistics IRCCS Istituto Romagnolo per lo Studio dei Tumori (IRST) “Dino Amadori” Meldola Italy; ^4^ Medical Oncology Unit IRCCS Istituto Romagnolo per lo Studio dei Tumori (IRST) “Dino Amadori” Meldola Italy; ^5^ Radiology Unit IRCCS Istituto Romagnolo per lo Studio dei Tumori (IRST) “Dino Amadori” Meldola Italy

**Keywords:** EGFR, extracellular vesicles, liquid biopsy, NSCLC, RNA

## Abstract

The mutational status of the epidermal growth factor receptor (*EGFR*) guides the stratification of non‐small cell lung cancer (NSCLC) patients for treatment with tyrosine kinase inhibitors (TKIs). A liquid biopsy test on cell‐free DNA is recommended as a clinical decision‐supporting tool, although it has limited sensitivity. Here, we comparatively investigated the extracellular vesicle (EV)‐RNA as an independent source for multidimensional and longitudinal *EGFR* profiling in a cohort of 27 NSCLC patients. We introduced and validated a new rapid, highly specific EV‐RNA test with wild‐type (WT) and mutant‐sensitive probes (E746‐A750del, L858R, and T790M). We included a cohort of 20 NSCLC patients with *EGFR* WT tumor tissues and systematically performed molecular EV‐RNA and circulating tumor DNA analyses with clinical data statistics and biophysical profiles of EVs. At the single‐patient level, we detected variegated tumor heterogeneity dynamics supported by combinations of driver *EGFR* mutations. EV‐RNA‐based mutation analysis showed an unprecedented sensitivity of over 90%. The resistance‐associated mutation T790M frequently pre‐existed at baseline with a gained EV‐transcript copy number at progression, while the general mutational burden was mostly decreasing during the intermediate follow‐up. The biophysical profile of EVs and the quantitative assessment of T790M revealed an association with tumor size determined by the sum of the longest diameters in target lesions. Vesicular RNA provides a validated tool suitable for use in clinical practice to investigate the dynamics of common driver *EGFR* mutations in NSCLC patients receiving TKIs.

## Graphical abstract

We applied a rapid and quantitative pipeline for detecting driver *EGFR* mutations in a retrospective cohort of NSCLC patients using circulating tumor DNA and extracellular vesicles' RNA (EV‐RNA) as independent longitudinal sources. The EV‐RNA provided sensitive and consistent dynamics of tumor heterogeneity with the potential to advance liquid biopsy tests in patients receiving tyrosine kinase inhibitors.

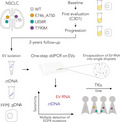

**Abbreviations**
ctDNA: circulating tumor DNA
EGFR: epidermal growth factor receptor
EV: extracellular vesicle
NSCLC: non‐small cell lung cancer
TKI: tyrosine kinase inhibitors

## 1 Introduction

Non‐small cell lung cancer (NSCLC), which accounts for about 85% of all lung cancers, is often diagnosed in advanced stages and requires systemic treatment [1]. In the Asian ethnicity, mutations in the epidermal growth factor receptor (*EGFR*) gene occur in ~ 50% of all lung adenocarcinomas. Meanwhile, in the Caucasian population, 15% of NSCLC patients harbor driver *EGFR* mutations, that is, deletions in exon 19 (E746_A750del) or point mutation in exon 21 (L858R). The introduction of first‐ and second‐generation tyrosine kinase inhibitors (TKIs) targeting EGFR (gefitinib, erlotinib, and afatinib) has improved outcomes in patients with EGFR‐mutant tumors [2, 3, 4]. The majority of patients, however, develops resistance after a median treatment time of 10–12 months. A secondary acquired *EGFR* mutation in exon 20, namely T790M, is associated with disease progression in 50–60% of cases [5]. Currently, the TKI osimertinib represents the third‐generation treatment for T790M‐positive patients [6]. Osimertinib showed superior efficacy over first‐generation TKIs in patients with identified EGFR‐activating mutations [7] and was recently approved as first‐line therapy in these subjects. Hence, testing for *EGFR* mutations is mandatory under treatment regimens to assess the presence of evolving resistance alterations. Repeated tissue biopsies are not always possible and virtually limited for the partial view of tumor heterogeneity, especially in cases with metastatic disease. Therefore, the refinement of molecular testing from a liquid biopsy is crucial to gain a non‐invasive screening, disease monitoring, and clinical decision‐supporting tool. Plasma circulating tumor DNA (ctDNA) represents a useful source to assess the tumor mutational status in the treatment course. Several studies already evaluated the suitability of ctDNA for detecting *EGFR* mutations [8, 9, 10] and, currently, international guidelines recommend the use of liquid biopsy in the clinical practice for NSCLC patients [11]. Several methodologies have been used for detecting *EGFR* mutation in ctDNA, including Amplification Refractory Mutation System [12, 13], droplet digital PCR (ddPCR) [14], and next‐generation sequencing [15]. Although ctDNA testing confirms a high specificity (~ 90%) [16], the sensitivity decreases to ~ 60% when matching liquid vs tissue biopsies [11]. From the biological point of view, this performance may rely on a certain threshold of tumor cellularity and/or specific mechanisms that enrich body fluids with cell‐free DNA, such as apoptosis, necrosis, phagocytosis, or active secretion [17]. Recent reports indicated that extracellular vesicles (EVs) could be a valuable source for *EGFR* testing [18, 19]. EVs are membranous vesicles massively secreted from the cells in biofluids and carry various coding and non‐coding RNAs, proteins, lipids, or metabolites mirroring the cells of origin [20]. Plasma EVs are heterogeneous in size and antibody‐based characterizations indicate a substantial derivation from leukocytes, platelets, erythrocytes, or endothelial cells populating blood and vessels, with a circumstantial contribution of different epithelial tumor cells [21, 22, 23]. Interestingly, the screening of exosomal RNA for *EGFR* mutations improved the overall sensitivity to 98% in combination with ctDNA in NSCLC patients [18]. However, we lack comparative studies on the differential contribution of EVs and ctDNA in longitudinal trials and the extent of feasibility through which EVs are applied for multi‐level screening. This pilot study involves longitudinal blood specimens, including baseline and follow‐up time points, from a selected cohort of NSCLC patients with *EGFR* mutation‐positive tumors. We systematically performed liquid biopsy testing including ctDNA and biophysically profiled plasma EVs obtained by nickel‐based isolation (NBI) [24, 25]. We applied a stringent cross‐validation pipeline to compare results obtained through EVs and cfDNA and a suitable workflow to screen for specific *EGFR* mutations directly using isolated EVs in a normalized, quantitative ddPCR assay.

At the single‐patient level, we detected a complex picture of tumor heterogeneity supported by combinations of driver *EGFR* mutations, sporadically seen also at ctDNA level, at the baseline, and two consecutive liquid biopsies. We found that the resistance‐associated mutation T790M was remarkably present in vesicular mRNA at baseline and increased during follow‐up, while the displayed quantitative mutational burden was generally decreasing during the applied treatments. We also tested EGFR‐positive EV sub‐populations to assess *EGFR* status and the feasibility of EV‐based multidimensional tests for sensitively monitoring patients receiving TKIs.

## 2 Materials and methods

### 2.1 Patients

Overall, 27 patients were enrolled from January 2015 to November 2017, as part of a prospective study conducted in IRST‐IRCCS on patients histologically confirmed with advanced NSCLC and positive to *EGFR* mutations. The study was approved by the Local Ethical Committee (CEIIV, No. 1680/2015); all patients gave their consent to participate in the study. All patients received a first‐line treatment with a first (either gefitinib or erlotinib) or second (afatinib) generation TKI. Treatment was continued until disease progression or unacceptable toxicity. Best clinical response to treatment with TKI was classified on the basis of interval computerized tomography (CT) scans as complete response, partial response, stable disease (SD), or progressive disease (PD) using the standard Response Evaluation Criteria in Solid Tumors version 1.1 [26]. Periodically, peripheral blood samples were withdrawn during treatment: at baseline, first clinical evaluation (C3D1), and progression. Blood samples were collected into EDTA‐containing tubes and centrifuged within 2 h to obtain plasma aliquots that were stored at −80 °C until DNA extraction or EVs isolation. At the time of progression, 6/27 (18%) patients underwent rebiopsy for tumor molecular characterization as part of clinical practice. Tumor data were obtained using medical and radiographic records; patient characteristics and follow‐up information included age, gender, smoking history, histology, information on death, response to first, and further lines of TKI treatments (full description in Table S1). The study conformed to the standards set by the Declaration of Helsinki.

### 2.2 Determination of EGFR mutations on tissue biopsy

Genomic DNA (gDNA) was extracted from hematoxylin and eosin‐stained 10‐μm sections of formalin‐fixed paraffin‐embedded (FFPE) tumor tissue, prepared at diagnosis and at progression when possible. Tumor area were selected by expert pathologists for containing at least 50% of tumor cells. Before DNA extraction, the tissue was deparaffinized in xylene and washed in 70% ethanol. DNA was isolated with the Gene All Tissue DNA Purification Kit (General Biosystem, Durham, NC, USA) and quantified by the Qubit fluorometer (Life Technologies, Carlsbad, CA, USA). Mutation analyses were performed in the Laboratory of Molecular Diagnostics at IRST‐IRCCS on 5 ng of extracted DNA by either single‐base extension through the Myriapod® Lung Status panel for nucleotide variants of genes associated with lung cancer (Diatech Pharmacogenetics, Iesi, Italy), combined with MALDI‐TOF mass spectrometry on the MassARRAY® System (SEQUENOM, San Diego, CA, USA), or by Pyrosequencing methodologies (Qiagen, Germantown, MD, USA). Verification of positive *EGFR* samples for the exon 19 deletion E746_A750del (c2235_2249del) was performed by using the Easy® EGFR Real‐Time PCR (Diatech Pharmacogenomics).

### 2.3 Detection of EGFR mutations on ctDNA isolated from plasma

Cell‐free DNA was extracted from 2 mL of plasma by using the QIAamp Circulating Nucleic Acid Kit (Qiagen) and stored at −80 °C until subsequent use. For each longitudinal time point, *EGFR* exon 19 deletion E746_A750del (c2235_2249del), L858R (c.2573T>G), and T790M (c.2369C>T) mutations were detected with Easy® EGFR Real‐Time PCR (Diatech Pharmacogenomics) on the Corbett Rotor‐Gene 6000 (Qiagen), which allows quantitative PCR (qPCR) co‐amplification of the mutated allele along with an endogenous control gene with sequence‐specific FAM and HEX probes, in a well‐defined WT/mutant allelic ratio, to obtain the detection of low fractions of the mutated allele in presence of high amounts of WT gDNA. At baseline, validation of *EGFR* mutations was performed by the QX200™ Droplet Digital™ PCR System (Bio‐Rad Laboratories, Foster City, CA, USA), by using the ddPCR™ Mutation Assays (EGFR E746_A750del, dMDS134490094; EGFR L858R, dMDS816628475; EGFR T790M, dHsaMDV2010019), already described above. The assays allow simultaneous detection and quantification of FAM‐mutant and HEX WT allele number of copies per mL. For both qPCR and ddPCR, 5 µL of ctDNA obtained by QIAamp (Qiagen) was amplified according to the manufacturer's protocol. Threshold was determined by the signal of no template, WT, and positive internal DNA controls. For ddPCR, at least three positive events were used as cutoff for false positivity. The ctDNA information was not available for all patients at every longitudinal blood withdrawal.

### 2.4 Nickel‐based isolation of extracellular vesicles from plasma

Extracellular vesicles were isolated from one mL of platelet‐poor plasma by using a previously described procedure [24]. Briefly, platelet‐poor plasma was obtained by centrifugation at 3000 ***g*** for 10 min at room temperature and diluted with PBS 1X to reduce the viscosity of the media (1 : 3 dilution). Then, positively charged beads (GE Healthcare, 17‐5268‐01) in suspension with NiSO_4_ (Sigma‐Aldrich, Darmstadt, Germany) were gently poured on the upper solution of the plasma to capture EVs and the solution was placed in orbital shaking for 30 min. Bead‐bound EVs were then recovered by gentle centrifugation at 600 ***g*** and eluted with a buffer designed to allow pH shifting and EV‐beads dissociation. After purification, EVs were measured by Tunable Resistive Pulse Sensing (TRPS) technology using qNANO instrument (Izon Science, Christchurch, New Zealand) upon calibration with CPC200 calibration particles (Izon Science) and NP250 nanopores (Izon Science A61484, A61489, A61531, and A61534) were used to characterize particles with a range diameter of 110–630 nm.

### 2.5 Detection of *EGFR* mutations on vesicular RNA

The molecular analyses on EVs and EV‐RNA were performed in blind.

An aliquot of EVs, resuspended in NBI‐elution buffer and quantified by TRPS, was used as template of a one‐step reaction with direct coupling of EV partitioning, reverse transcription, and pre‐amplification with specific primers [24]. *EGFR* mutations were detected by the QX200™ Droplet Digital™ PCR System (Bio‐Rad Laboratories), by using the ddPCR™ Mutation Assays (EGFR E746_A750del, dMDS134490094; EGFR L858R, dMDS816628475; EGFR T790M, dHsaMDV2010019). The method was optimized on 10^5^ polydisperse EVs to obtain at least 15 000 droplets in each assay. Sensitivity of the method was first set up on control cell lines: NCI‐H1650 [ATCC® (Manassas, VA, USA) CRL‐5883™] for E746‐A750del and NCI‐H1975 (ATCC® CRL‐5908™) for L858R and T790M mutations. Specificity of the method was assessed by testing the ddPCR™ Mutation Assays on EVs isolated from plasma of 34 healthy donors and from plasma of 20 cases EGFR WT. Signal from liquid biopsy, as measured by ddPCR™ Mutation Assays, was considered positive when at least one FAM‐positive droplet was detected every 10^5^ EVs counted. Threshold was determined by the signal of no template and one ng of RNA control derived from cell lines positive to the mutations tested, extracted using TRIzol® (Sigma‐Aldrich) Reagent, according to the manufacturer's instructions. RNA extracted from cells was quantified at NanoDrop™ (Thermo Scientific, Waltham, MA, USA) Spectrophotometer. EV isolation was not possible for all patients at every longitudinal blood withdrawal for insufficient starting material. The number of mutated and WT mRNA copies was calculated using Bio‐Rad QuantaSoft™ Analysis Pro (QuantaSoft AP, Hercules, CA, USA) Software. Data were normalized on the number of particles counted per mL of plasma.

### 2.6 AlphaScreen assay combined with immunoprecipitation and droplet digital PCR

The presence of EGFR on the surface of EVs was analyzed in a range of EVs isolated by NBI from the plasma of NSCLC patients along with the longitudinal study, previously characterized by TRPS. The screen assay was carried out in 384‐OptiPlate (Perkin Elmer, Waltham, MA, USA) in a final volume of 20 μL using 10^5^–10^6^ vesicles as template, 15 μg·mL^−1^ of Nickel Chelate AlphaLISA Acceptor Beads (Perkin Elmer), 10 μg·mL^−1^ of AlphaScreen Streptavidin Donor beads (Perkin Elmer) and 20 ng of biotinylated anti‐EGFR antibody (OriGene, Rockville, MD, USA). Serial dilutions of the antibody were previously tested to determine the hook point in PBS 1X solution. Alpha counts were revealed by the EnSpire instrument (Perkin Elmer) after 60 min of plate incubation in the dark at room temperature. Once assessed EGFR expression on EV surface, vesicles were resuspended in 500 µL of PBS 1X, incubated with 100 ng of biotinylated anti‐EGFR antibody and Streptavidin Dynabeads (Thermo Fisher Scientific, Waltham, MA, USA). Streptavidin magnetic Dynabeads were used to precipitate EGFR‐positive EVs and proceed with TRIzol® Reagent, according to the manufacturer's instructions. Given the insufficient amount of vesicles from patients to replicate the experiment using IgG‐negative control antibody, we performed the procedure in parallel using 10^6^ EVs previously isolated from eight healthy subjects. EGFR‐positive EVs of NSCLC patients eluted by streptavidin Dynabeads and EVs isolated from healthy subjects were used as the template of ddPCR reactions and tested for the presence of EGFR mutations by NBI‐ddPCR on the QX200™ Droplet Digital™ PCR System (Bio‐Rad Laboratories), using the ddPCR™ Mutation Assays (EGFR E746_A750del, dMDS134490094; EGFR L858R, dMDS816628475; EGFR T790M, dHsaMDV2010019) according to the protocol previously applied to bulk EVs.

### 2.7 Validation of *EGFR* mutations on EV transcripts by Sanger sequencing

Epidermal growth factor receptor variants found by NBI‐ddPCR on EV‐RNA were confirmed by Sanger sequencing. A total number of 10^5^ EVs isolated from NCI‐H1975 and NCI‐H1650 were processed for the detection of both L858R and T790M point mutations and E746_A750del, respectively. RNA encapsulated in lysed EVs was subjected to retro transcription and to a double PCR amplification, to be later analyzed by Sanger sequencing. Primers used for PCR amplification (Table S4) were designed to target the region surrounding the candidate mutations. In detail, PCR reactions were performed in volumes of 50 μL composed of 2X MyTaq Mix (Bioline Reagents Ltd., London, UK), 0.8 µm primers, 5 U per rxn of WarmStart® RTx Reverse Transcriptase (New England BioLabs, Ipswich, MA, USA) ddH_2_O and 20%/v template EVs. A Bio‐Rad C1000 thermal cycler (Bio‐Rad) was used with the following thermocycling conditions: 20 min at 55 °C, 1 min at 95 °C, followed by 30 cycles of 15 s at 95 °C and then at 58 °C, finally 10 s at 72 °C. Subsequently, PCR products were further amplified in a second PCR reaction of 50 µL without retro transcriptase with the following composition: 2X MyTaq Mix, 0.8 µm of the previous primer sets, ddH_2_O and 30%/v template PCR products of the first reaction. Thermocycling conditions did not change with respect to the first reaction, except for the absence of the retro transcription step at 55 °C for 20 min. The products were then checked for the presence of unspecific sequences on 2% TAE‐agarose gel stained with SYBR Green Stain (Life Technologies, Carlsbad, CA, USA) through gel electrophoresis on a Bio‐Rad gel electrophoresis system (Bio‐Rad). ChemiDoc XRS imaging system (Bio‐Rad) was used for the detection of end‐point PCR products. Amplification products were then purified using the NucleoSpin Gel and PCR Clean‑up kit (Macherey‐Nagel, Düren, Germany) and measured by NanoDrop 2000 Spectrophotometer (Thermo Scientific). Sequencing was performed by Eurofins Genomics (Ebersberg, Germany) using both forward and reverse sequencing PCR primer sets. Electropherograms were analyzed on chromas Software 2.6.6 (Technelysium, Brisbane, Australia).

### 2.8 Statistical analysis

Statistical evaluations were performed by using graphpad prism software (San Diego, CA, USA) version 8. All tests used are listed in the main text and in the figure legends. Concordance between mutations detected in ctDNA, EV‐RNA, and tissue biopsy was calculated in the subset of patients with matched plasma and tissue samples available only. Longitudinal changes in the EV ratio were measured by dividing the number of total EVs of the consequent clinical time point (divided termed) by the antecedent clinical time point (divisor). Given the impossibility to divide the terms in the ratio by zero (cases where no mutated copies were detected), the quantitative change of T790M levels in EV‐RNA was calculated by subtracting the number of mutated copies at the antecedent clinical time point from the number of mutated copies at the consequent clinical time point, as detected by NBI‐ddPCR (expressed in 10^10^ copies·mL^−1^). In the same way, the difference in the relative change in the amount of T790M to activating EGFR mutations was calculated by subtracting the number of copies of activating EGFR mutations (E746_A750del + L858R) from the number of mutated T790M copies, at the corresponding clinical time point. Results were considered statistically significant when *P*‐value was < 0.05 (*), < 0.01 (**), and < 0.001 (***).

## 3 Results

### 3.1 Workflow for ultrasensitive detection of *EGFR* mutations in blood‐circulating EVs

**Fig. 1:**
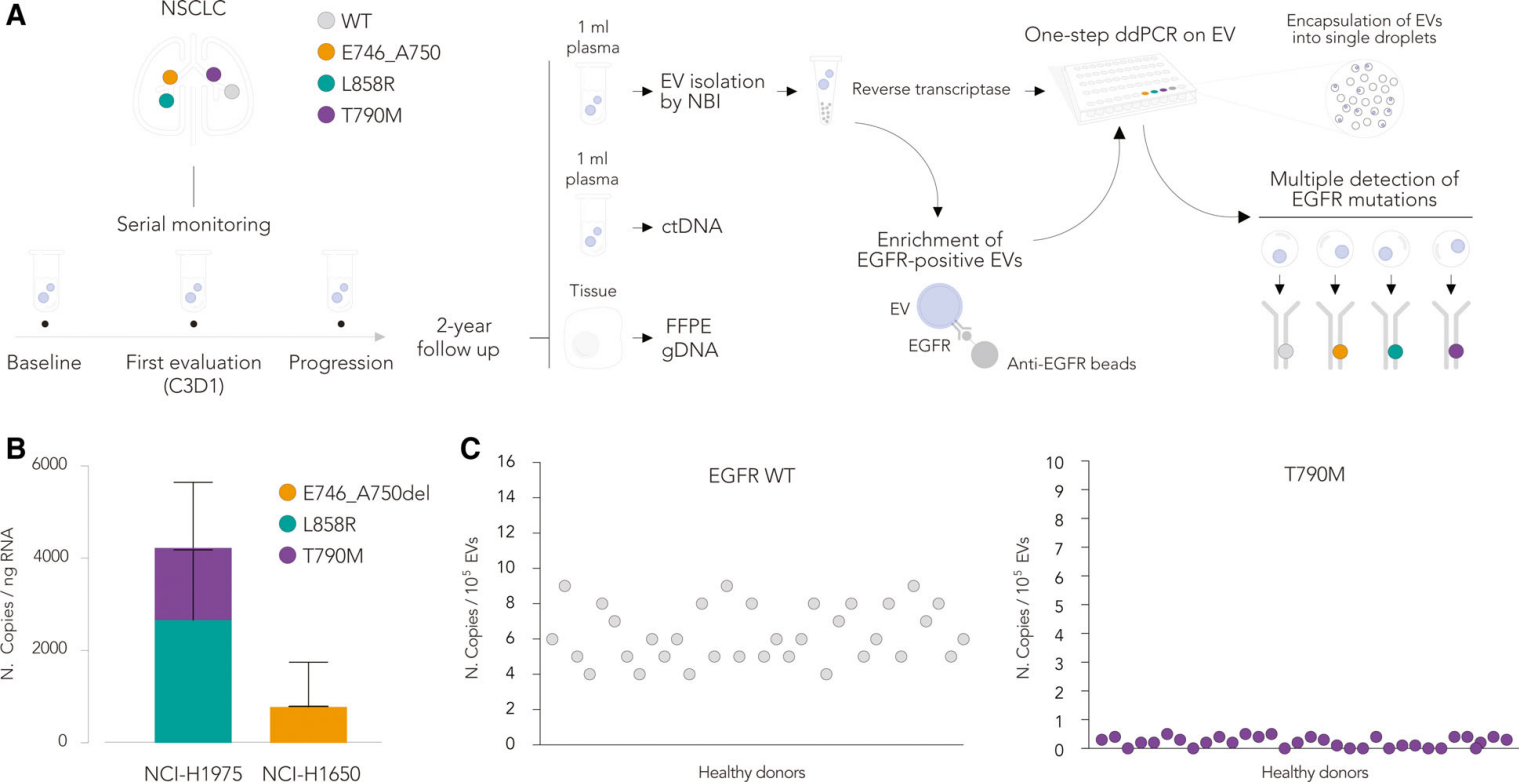
Experimental validation of the NBI‐ddPCR method. (A) Overview of the NBI experimental protocol and study workflow for high‐sensitivity detection of *EGFR* activating and resistance mutations on plasma clinical samples. EVs were isolated from patient samples collected at baseline, first clinical evaluation (C3D1), and progression, and compared with available ctDNA and tissue biopsy analysis. Plasma‐isolated EVs were directly analyzed through one‐step ddPCR with mutation‐specific fluorescent probes that could detect in a single assay the presence of both the WT and mutated *EGFR* mRNAs. Prior to mutation detection by ddPCR, a fraction of the isolated EVs were subjected to immunoprecipitation with anti‐EGFR antibody beads to potentially enrich for tumor‐derived EVs. (B) Validation of the one‐step ddPCR EGFR mutation detection assay on EVs isolated from NSCLC cells by NBI (*n* = 3, mean with SD). No statistical test was applied for comparison. (C) Validation of the one‐step ddPCR EGFR mutation detection assay on EVs isolated from plasma samples of healthy individuals by NBI. Number of mutated *EGFR* copies on EV‐RNA obtained by NBI‐ddPCR is represented as the number of copies/10^5^ EVs.

To test the contribution of EVs as an independent source for liquid biopsy, we applied NBI as a low‐cost and rapid procedure to capture EVs through the net negative charge of membranes and to subsequently elute them in a physiological pH buffer [24, 25]. We designed an NBI‐ddPCR procedure starting from one milliliter of plasma samples from a selected cohort of 27 NSCLC patients (Table 1 and Table S1). Patients harbored *EGFR* activating mutations, as detected by tumor biopsy, and were treated with anti‐EGFR TKIs in the first line. Longitudinal peripheral blood samples were collected at the first clinical evaluation (C3D1) and at progression. We focused our study on the three most recurrent *EGFR* alterations, including the E746_A750 deletion (c.2236_2249del), the L858R (c.2573T>G), and the T790M (c.2369C>T) point mutations, as depicted in Fig. 1A. We used NBI to rapidly isolate EVs as already described, therefore preserving EV integrity and the single‐particle distribution in solution [24]. EVs were systematically characterized by TRPS using NP250 nanopores. Next, we designed a TaqMan based‐One‐Step droplet digital™ PCR (ddPCR™) assay to analyze the RNA carried by a defined number of input vesicles (Fig. 1), considered as a parameter to normalize the abundance of detected transcripts. The assay employed mutation‐specific probes in a competitive reaction to detect both mutant and WT *EGFR* transcripts. We optimized the assay using the total RNA isolated from NCI‐H1975 cells (bearing L858R and T790M mutations) and NCI‐H1650 cells (expressing a heterozygous E746_A750del in *EGFR*). We were able to simultaneously detect individual *EGFR* mutations (FAM fluorophore) in both cell lines, as well as transcripts containing the WT *EGFR* sequence (HEX fluorophore), starting from as low as one ng of total RNA (Fig. 1B). We performed titration analyses using NBI‐isolated EVs and the EV‐RNA as input material to validate the test's high specificity and sensitivity *in vitro*, which required as low as 10^4^ vesicles to detect the mutation‐specific signal (Fig. S1A). We reproducibly detected *EGFR* mutations at EV‐RNA level with an average ratio of 1 : 100 compared to the number of copies of matching transcripts in the origin cells. To assess the rate of false positives during the amplification of rare events, we additionally performed two sequential PCR amplification. The Sanger sequencing finally confirmed the straightness of the test according to EVs of different derivation (Fig. S1B). We applied the NBI‐ddPCR workflow to EVs isolated from 1 mL of plasma obtained from 34 healthy subjects (Fig. 1C). Upon measuring the concentration of recovered EVs by TRPS with an NP250 nanopore (Fig. S2A), we detected the WT *EGFR* transcript with an average of 6 copies every 10^5^ EVs; at the same time, the test on T790M gave a threshold below one copy (Fig. 1C).

**Table 1:** Patient cohort (*n* = 27). Percentage may exceed 100% due to presence of double mutations.

Characteristic	No. of patients (%)
Age, years	
Median	69
Range	37–86
Sex	
Female	19 (70)
Male	8 (30)
Smoking habit	
Current	4 (15)
Former	7 (26)
Never	16 (59)
First‐line TKI therapy	
Gefitinib	9 (33)
Erlotinib	6 (22)
Afatinib	12 (45)
EGFR mutation tumor diagnosis	
E746_A750del	12 (45)
L858R	9 (33)
Other	7 (26)
Tumor rebiopsy	
T790M negative	5 (18)
T790M positive	1 (4)
ND	21 (78)

### 3.2 Longitudinal assessment of *EGFR* status in cell‐free DNA and EV‐RNA

**Fig. 2:**
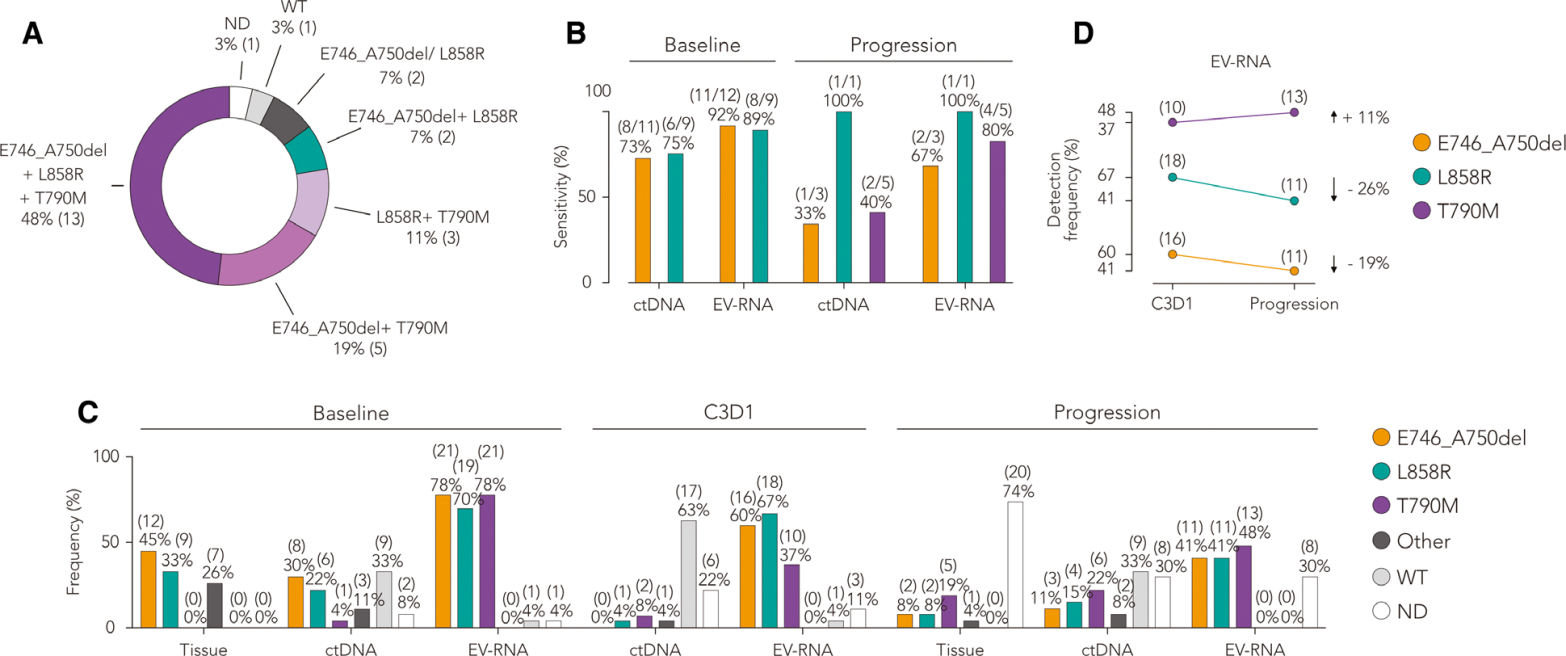
Detection of *EGFR* mutations in tumor tissue and sensitivity by liquid biopsy. (A) Rate of co‐occurrence of multiple mutations (activating and resistance) at baseline, as measured by NBI‐ddPCR (numbers in parenthesis indicate the relative number of cases over total, *n* = 27; WT indicates samples negative for any type of mutation tested; percentage values have been rounded). (B) Comparison of liquid biopsy by EV‐RNA NBI‐ddPCR with canonical ctDNA (qPCR) analysis for the detection of E746_A750del, L858R, and T790M mutations, shown as percentage of concordance (referred as sensitivity in detecting positivity to *EGFR* mutations) with the corresponding tissue biopsy (numbers in parenthesis indicate the number of patients that had matched results between tissue and liquid biopsy, *n* = 27). No statistical test was applied for comparison. (C) Frequency of patients with *EGFR*‐activating and T790M resistance mutations detected in tissue and liquid biopsy, at baseline, first clinical evaluation (C3D1), and progression (other mutations: L747_S752del, E746‐S752>V, E746‐T751del, G719S, L833V, L861Q, L858M, exon 20 insertion; WT indicates samples negative for any type of mutation tested). Only E746_A750del, L858R, and T790M were tested on EV‐RNA following NBI‐ddPCR. Sum of percentages may exceed 100% of total because of rounding and concomitant presence of multiple mutations in the same patients (*n* = 27; ctDNA values obtained by mean of qPCR). No statistical test was applied for comparison (D) Difference in detected fractions of positive cases with EGFR‐activating and T790M resistance mutations from C3D1 to progression, as measured by EV‐RNA liquid biopsy (numbers in parenthesis indicate the relative number of cases over total, *n* = 27; percentage values have been rounded).

Six patients (22%) had tissue rebiopsy at the time of progression to verify the T790M resistance mutation (Table 1 and Table S1). At baseline, qPCR results on cfDNA matched tissue biopsy profile with a specificity of ~ 70%, with T790M identified in 4% of cases (Fig. 2). Qualitatively, the concordance between tissue biopsy and cfDNA analyses was 73% (8/11) for the E746_A750del and 75% (6/8) for the L858R. At progression, three cases only (out of eight) remained positive to the E746_A750del, while four (out of six) were still positive to the L858R. As expected from prolonged treatments, T790M was identified in five new more patients (#1, #2, #9, #25, #26), six in total from baseline. From C3D1 to progression, one case (#16) resulted negative for the mutation, and three patients (#9, #25, #26) showed positivity to both L858R and T790M, along with case 14; Patient 1 displayed both E746_A750del and T790M mutations.

By NBI‐ddPCR on EV‐RNA, we detected at baseline the *EGFR* E746_A750del and L858R mutations in 78% and 70% of cases, respectively. In line with tissue biopsy data, the most represented mutation was the *EGFR* E746_A750del, with an average copy number of 3.64*10^10^/mL vs 0.03*10^10^/mL of L858R in ddPCR. Fifty‐five % of cases (*n* = 15) showed co‐occurrence of both driver mutations, including 48% of patients (*n* = 13) that showed co‐presence of both drivers and resistance T790M mutation (Fig. 2A). Compared with tissue biopsy at baseline, the sensitivity was 92% for E746_A750del and 89% for L858R (Fig. 2B), where cases #20 and #21 were not informative for this test, being L858R detected in #21 by ddPCR on cfDNA. Unexpectedly, 78% of cases resulted positive to T790M at baseline (Fig. 2C), matching only one case (#14) on tissue DNA and detected almost exclusively in EVs with a remarkable 1.65*10^10^ copies·mL^−1^ on average.

The comparative study of longitudinal liquid biopsies indicated that the frequency of not determined (ND) mutations was 8% for cfDNA and 4% for EV‐RNA (Fig. 2C). At C3D1, the percentage of ND mutations was 14% for cfDNA. In comparison, it decreased to 8% for EV‐RNA, demonstrating a better sensitivity of vesicle transcripts in a context where the number of mutations decreased in all biological sources due to TKIs treatment. At progression, this trend reversed in cfDNA, with a gain of 15%, 11%, and 14% of cases positive to E746_A750del, L858R, and T790M, respectively. EV‐RNA showed in turn a reduced number of cases positive to E746_A750del (−19%) and L858R (−26%) from C3D1 to progression, with a remarkable gain of 11% of new cases positive to T790M (Fig. 2D).

**Fig. 3:**
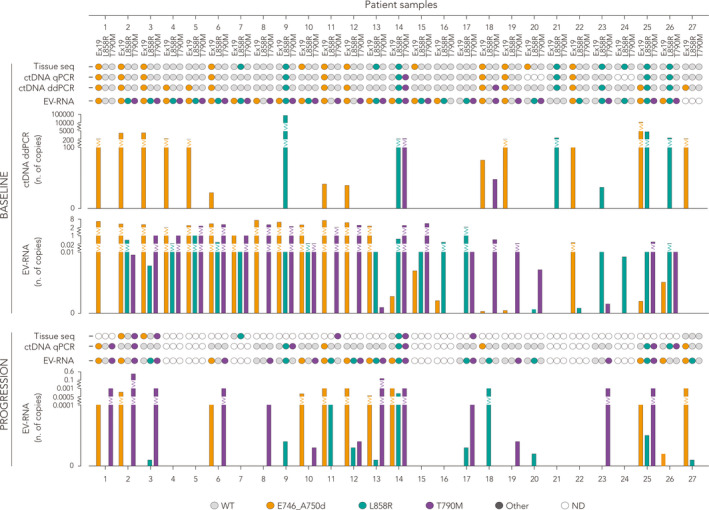
Tumor heterogeneity and high frequency of T790M mutation by testing *EGFR* in ctDNA and EV‐RNA. Intra‐patient heterogeneity of *EGFR* mutations and levels of T790M detected by liquid biopsy analysis at baseline and progression. At baseline, analysis of ctDNA was performed by both qPCR and ddPCR. Only samples tested for E746_A750del, L858R, and T790M are included (*n* = 27; WT indicates samples negative for any type of mutation tested). Number of mutated *EGFR* copies on EV‐RNA obtained by NBI‐ddPCR is represented as linear multiple of 10^10^ copies·mL^−1^.

The panel of detected *EGFR* mutations at a single‐patient level, either in tissue and liquid biopsies, is displayed in Fig. 3. Interestingly, patients #10 and #14, positive to E746_A750del, and patient #12, positive to L858R at baseline, resulted negative at the intermediate C3D1 and then again positive at progression. Conversely, cases negative to L858R (#1, #8, #11, #18) at baseline gained the mutation later at C3D1, and cases #11 and #18 maintained the positivity at progression. One patient (#12) was tested positive to L858R for the first time at progression. Notably, the percentage of patients positive to A746_750del and L858R at baseline (57%), decreased to 54% at C3D1 and to 31% at progression.

By EV‐RNA analysis, we were also able to measure the amount of WT *EGFR* transcript in circulation. In detail, we examined the patient samples from baseline to progression and found that 98.7% of EV transcripts expressed *EGFR* (Table S2 indicates patients as WT for the mutation highlighted in the column and the combination of *EGFR* mutations detected by the assay). As compared to healthy donors, the blood of NSCLC patients was also much more enriched in non‐mutated *EGFR* copies (Fig. 1C and Fig. S2B), possibly correlating with the presence of tumor‐derived EVs or vesicles deriving from other cells with an ‘altered/activated’ metabolism. To biologically validate the results described, we investigated the method's performance in assessing the EV‐*EGFR* status in a separate cohort of 20 NSCLC patients stratified as *EGFR* WT both in tissue biopsy and cfDNA (Fig. S3). As shown in Fig. S3 (see particle size and concentration in Fig. S3A), we detected none of the *EGFR* mutations screened (Fig. S3B), with the exception of two cases (#4 and #20) that displayed E746_A750del in EV‐RNA (Fig. S3C). However, the copy number (respectively 0.042*10^10^ and 0.043*10^10^/mL) is neglectable compared to the average number of E746_A750del in the longitudinal cohort. Further analysis on tissue DNA by Easy® EGFR Real‐Time PCR (Diatech Pharmacogenomics) confirmed the absence of mutation in these two cases.

Overall, the EV‐RNA‐based *EGFR* analysis revealed a complex tumor heterogeneity dynamic with unprecedented resolution, implicating it as a potential clinical decision‐supporting tool.

### 3.3 Multidimensional vesicular profile and quantitative assessment of *EGFR* RNA

**Fig. 4:**
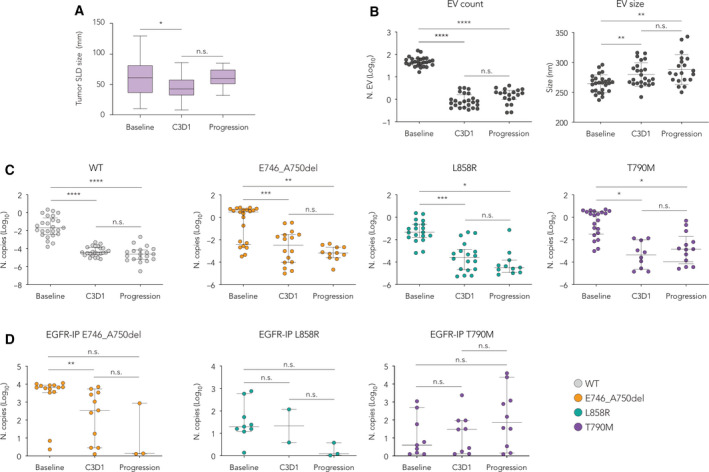
Longitudinal analysis of *EGFR* mutations in NSCLC patients revealed by vesicular RNA. Longitudinal measurements of EV profiles were taken at baseline, first clinical evaluation (C3D1), and progression, alongside to tumor dimensional changes. (A) Box and whiskers distribution (median, min to max) of tumor size determined by the sum of longest diameters (SLD) and compared between baseline and C3D1 (*n* = 23; *P > 0.05, Wilcoxon signed‐rank test, two‐tailed) and between progression and C3D1 (*n* = 22; n.s., not significant, Wilcoxon signed‐rank test, two‐tailed). (B) Total EV count, represented as Log10 conversion of 10^10^ copies per mL of plasma, and EV size quantified by TRPS. (C) Number of mutated *EGFR* copies on EV‐RNA as measured by NBI‐ddPCR and represented as Log_10_ conversion of 10^10^ copies·mL^−1^ (WT indicates samples negative for any type of mutation tested). (D) Number of mutated *EGFR* copies on EV‐RNA as measured by NBI‐ddPCR, represented as Log10 conversion of 10^5^ copies·mL^−1^, and quantified upon immunoprecipitation (IP) against the membrane EGFR protein on isolated EVs. Scatter plots of individual value in (B–D) are represented as mean with SD (*P > 0.05, **P < 0.005, ***P < 0.001, ****P < 0.0001; n.s: not significant; Wilcoxon signed‐rank test, two‐tailed).

The tumor size was determined by the sum of longest diameters (SLD) of all target lesions by using the Response Evaluation Criteria In Solid Tumors (RECIST) [26] and was compared between baseline and C3D1 (*n* = 23) or between C3D1 and progression (*n* = 22). We observed a statistically significant reduction of SLD at C3D1 (Fig. 4A). EVs isolated by NBI were characterized in terms of relative abundance and size distribution by TRPS, using NP250 nanopores in a calibrated range of size between 120 and 800 nm. The average concentration of EVs at baseline was 52.8*10^10^ particles per milliliter of plasma. The treatment with TKIs was associated with a significant decrease of EVs' abundance (average of 1.19*10^10^/mL) at C3D1 that invariably persisted at progression (average of 1.74*10^10^/mL). Conversely, the mean diameter of vesicles was not affected throughout the longitudinal study, although a not significant, opposite trend could be observed (Fig. 4B).

By comparing the number of detected copies upon normalization on the number of vesicles, we observed that *EGFR* WT, as well as mutant transcripts, was considerably lower at C3D1 compared to baseline (Fig. 4C; *P* < 0.005, Wilcoxon paired test). We also noticed a consistent decrease of relevant mRNAs with a scattered trend inversion between C3D1 and progression in T790M (Fig. 4C). At progression, we observed an increased number of patients positive to T790M (13/27) compared to the first clinical evaluation (10/27). Notably, in most cases, the identification of T790M was previously detected in EV‐RNA at diagnosis (Figs 2 and 3, cases #2, #6, #8, #10, #12, #14, #23, #25). Overall, these data indicate a biological specificity in the ultrasensitive detection of *EGFR* mutations under testing, especially for T790M.

Considering the complex picture of *EGFR* status and the high frequency of mutated transcripts, we explored the NBI‐ddPCR pipeline in selected EV sub‐populations, virtually deriving from tumor cells by assuming the presence of EGFR receptor on their surface (Table S2). To this end, 10^4^ to 10^5^ NBI‐isolated EVs were diluted in 500 µL of PBS and incubated with a biotinylated anti‐EGFR antibody. Streptavidin Dynabeads were then used to precipitate the complexes and proceed with the RNA extraction from vesicles exposing EGFR on their surface (Table S3). Given the insufficient number of vesicles from patients to replicate the experiment using IgG‐negative control antibody, we performed the procedure in parallel using 10^7^ EVs previously isolated from eight healthy subjects. Strikingly, we still detected *EGFR* mutations by ddPCR, but we identified a lower number of positive cases than in bulk EVs. Almost 50% of cases previously detected resulted as not informative, particularly for E746_A750del, despite an interesting sensitivity for T790M, which displayed a more linear increase from baseline to progression (Fig. 4D). Nevertheless, these data suggest that an EV‐targeted strategy could be successfully explored for detecting specific, co‐segregating somatic alterations.

### 3.4 Correlation of molecular profiling with tumor progression and patient outcome

**Fig. 5:**
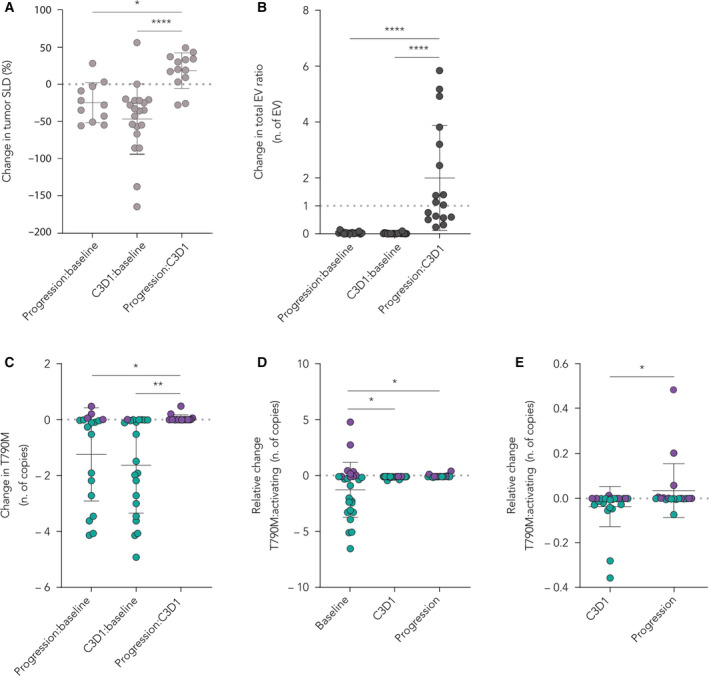
Longitudinal changes of tumor vesicular burden. (A) Percentage changes in tumor size determined by the SLD according to RECIST 1.1 criteria, compared between baseline to progression vs first clinical evaluation (C3D1) to progression (*n* = 11 vs *n* = 13; *P > 0.05, one‐way ANOVA with Tukey's comparison test) and between baseline to C3D1 vs progression to C3D1 size variation (*n* = 20 vs *n* = 13; ****P < 0.0001, one‐way ANOVA with Tukey's comparison test). (B) Change in the ratio of total EV number per mL of plasma, comparing the variation between baseline to progression (*n* = 18) vs C3D1 to progression (*n* = 17) and between baseline to C3D1 (*n* = 23) vs progression to C3D1 (*n* = 17), (****P < 0.0001; one‐way ANOVA with Tukey's comparison test). (C) Change in the amount of T790M from baseline to progression (*n* = 18), baseline to C3D1 (*n* = 21), and C3D1 to progression (*n* = 17), as measured by the variation in the total number of mutated EV‐RNA copies detected by NBI‐ddPCR (expressed as 10^10^ copies·mL^−1^). Patients in which the quantitative T790M change is low are in green and patients in which the quantitative T790M change is high are in violet (*P < 0.05, **P < 0.005; one‐way ANOVA with Tukey's comparison test). (D) Relative changes of T790M to activating mutations at baseline (*n* = 26), first clinical evaluation C3D1 (*n* = 24), and progression (*n* = 19), measured as the variation in the number of T790M mutated copies over the sum of both E746_A750del and L858R number of copies as detected by NBI‐ddPCR (expressed as 10^10^ copies·mL^−1^). Patients with low T790M:activating mutation ratio are in green and patients with high T790M:activating mutation ratio are in violet (*P < 0.05, Wilcoxon signed‐rank test). (E) Same as (D) with a close‐up comparison of the ratio change of T790M to activating mutations from C3D1 to progression (*n* = 17; *P < 0.05, paired two‐tailed *t*‐test). Graphs are represented as mean with SD.

Clinical information was available for all patients in the cohort (Table S1). Albeit the cohort statistics of this retrospective study limits the identification of predictive parameters, we explored possible associations between the data derived from EV analysis and clinical features of NSCLC patients. Overall, we observed a parallel change in circulating EVs and variation in the tumor volume (SLD). In particular, the increase of 24% in SLD from C3D1 to progression (Fig. 5A, *P* < 0.001 compared to C3D1:baseline and *P* = 0.02 compared to progression:baseline, one‐way ANOVA, and Tukey's multiple comparison test) was paralleled by a remarkable escalation in the number of EVs of most patients from C3D1 to progression (median twofold increase in the total EV count, *P* < 0.001, one‐way ANOVA and Tukey's multiple comparison test, Fig. 5B). Furthermore, we observed an equivalent increment in the copy number of T790M vesicular transcripts (relative T790M ratio between clinical time points) from C3D1 to progression (median increase of 0.04*10^10^ copies·mL^−1^). By contrast, the average number of EV‐RNA T790M copies was generally low at baseline and soon after initiating the TKI treatment (Fig. 5C, *P* = 0.02 for progression: C3D1 vs progression:baseline, *P* = 0.002 for progression/C3D1 vs C3D1/baseline, one‐way ANOVA, and Tukey's multiple comparison test). Similarly, at baseline the relative change (difference in the number of mutated copies per mL of plasma) between T790M to EGFR activating mutations (E746_A750del plus L858R), as measured on the circulating EV‐RNA, was considerably small, indicating that most patients had a lower amount of EVs that carried the T790M mutation; this disproportion progressively diminished going from pre‐treatment to C3D1 and to progression, with a shift toward an augmented number of patients having more copies of EV‐RNA T790M than E746‐A750/L858R in their blood when tumor progressed (Fig. 5D, *P* = 0.02 for C3D1 vs baseline, *P* = 0.02 for progression vs C3D1, one‐way ANOVA, and Tukey's multiple comparison test). In particular, the difference in the relative change of T790M copies·mL^−1^ over activating mutations was significantly higher at progression compared to C3D1 (Fig. 5E, *P* = 0.04, paired *t*‐test), suggesting a preferential post‐treatment selection of T790M‐containing tumor sub‐clones, more evident when tumors progress further.

**Fig. 6:**
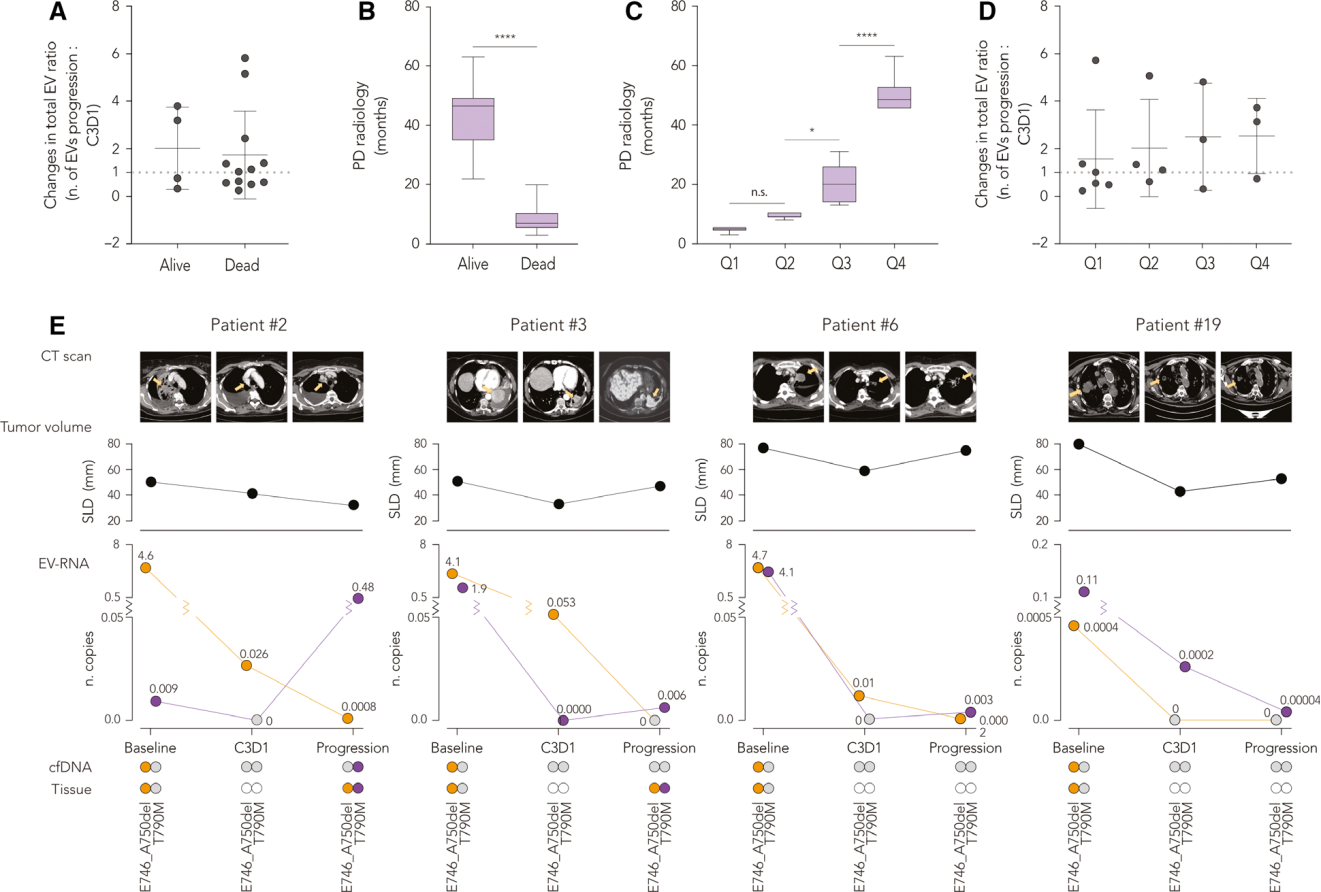
Association of NBI‐ddPCR analysis with clinical features and patient outcome. (A) Distribution of the changes in the ratio of total number of EVs between the first clinical evaluation (C3D1) to progression in relation to patient survival status. Differences between the two groups are not significant (unpaired two‐tailed *t*‐test; data are represented as mean with SD). (B) Box and whiskers distribution (median, min to max) of radiologic PD, defined by RECIST 1.1, in association with the survival status (Alive, *n* = 8; Dead, *n* = 14; ****P < 0.0001, unpaired two‐tailed *t*‐test). (C) Box and whiskers representation (median, min to max) of radiologic PD, defined by RECIST 1.1, as divided into quartile distributions of groups of patients (Q1, *n* = 7; Q2, *n* = 5; Q3, *n* = 5; Q4, *n* = 6; n.s., not significant, *P < 0.05, ****P < 0.0001; one‐way ANOVA with Tukey's comparison test; data are represented as mean with SD). (D) Distribution of the changes in the ratio of total number of EVs between the first clinical evaluation (C3D1) to progression in the four radiologic PD quartile groups as defined in (C). Differences in the four groups are not significant (one‐way ANOVA with Tukey's comparison test). (E) Representative CT scan images of selected patients at the three clinical time points with parallel detection of the driver E746_A750del (orange circles) and the resistance T790M (purple circles) EGFR mutations in liquid biopsy (EV‐RNA and ctDNA), as compared to tissue analysis. The largest target lesion is pointed out (yellow arrows) in the upper panel, while the volume of all lesions is represented as the SLD. Analysis of cfDNA was performed by qPCR; number of mutated EGFR copies on EV‐RNA obtained by NBI‐ddPCR is represented as multiple of 10^10^ copies·mL^−1^. WT: wild‐type samples for the indicated mutation.

In the majority of cases, the overall number of EVs measured at progression was higher compared to C3D1 (fold change threshold set at 1, Fig. 6A). These patients had a much shorter progression‐free survival, as defined by the CT analysis to radiologically determine the PD, which was on average of 8.2 months (Fig. 6B, *P* < 0.0001, unpaired *t*‐test). As a bottom line, the window from C3D1 to progression showed a slight tendency to a reduced EV release in the circulation of patients with worse outcomes, although not significant in unpaired *t*‐test. Based on the radiological PD, we divided the patients' cohort into four distinct groups (quartile distribution), pointing out the sharp difference in the fraction of patients that had longer PD, which included those patients still alive at the end of the study (Fig. 6C, *P* < 0.0001, one‐way ANOVA and Tukey's multiple comparison test). This PD distribution was partially mirrored by a progressive slope (statistically not significant) in the ratio of EVs released from C3D1 to progression (Fig. 6D). These observations might indicate that, during disease progression, a concomitant boost in EV release could be associated with the tumor masses that gradually expanded after C3D1 (Fig. 5B).

We selected four representative cases that displayed divergent patterns of E746_A750del and T790M mutations (Fig. 6E). In general, the absolute amount of EVs and copies of *EGFR* activating mutation progressively reduced following treatment, along with a partial decrease of the SLD size. In most patients, although levels of EV‐T790M were already high at baseline, we observed a quantitatively different increment of T790M copies after C3D1. In Patient #2, levels of EV‐E746_A750del were significantly elevated at the beginning and decreased throughout the therapy, while the T790M, which was undetectable at C3D1 (*n*.copies·mL^−1^ = 0), peaked at progression. This patient was a nonsmoker, treated with erlotinib, who developed radiological progression after 13 months, and for whom the NBI‐ddPCR test reflected the diagnosis obtained by tissue biopsy and cfDNA. Similar to Patient #2, Patient #3 and #6 showed a massive reduction of E746_A750del plasma copies released into EVs, and in both cases, the E746_A750del was co‐present with the T790M mutation. As for Patient #2, the levels of vesicular T790M drastically decreased soon after TKI treatment (accompanied by a reduction of the tumor masses) and emerged when the disease progressed, indicating this mutation as the predominant molecular link of resistance in these patients. Both Patients #2 (a nonsmoker) and #3 (a former smoker) developed radiological progression quite late, at 31 and 45 months, respectively, after being both treated with afatinib; these patients were still alive at the end of the study. Interestingly, for Patient #19, the signal from NBI‐ddPCR revealed that the copies of T790M in circulation at baseline were much more abundant compared to the activating mutation. In this patient, both EV‐RNA signals drastically decreased during disease progression (E746_A750del was absent in circulating EV at C3D1 and progression); conventional analysis on cfDNA resulted negative for the T790M at all clinical time points. Patient #19 was a former smoker, treated with afatinib in the first line, who died after 11 months of PD response. As shown in these examples, conventional cfDNA liquid biopsy often failed in detecting the presence of the EV‐E746_A750del in circulation, as well as co‐occurrence with the T790M (confirmed by tissue biopsy in patients #2).

## 4 Discussion

The usefulness of liquid biopsy in detecting and monitoring *EGFR* mutation in NSCLC is now widely recognized and routinely used in clinical practice, as indicated by national and international guidelines [11]. At baseline, liquid biopsy could be useful in circumstances where: (a) no sufficient tumor material is available, (b) only bone biopsy is available with difficulties to perform molecular analysis, and (c) the patient status does not permit invasive procedures to obtain biological material. For the assessment of *EGFR* status during TKI therapy, liquid biopsy testing allows for improved estimation of tumor heterogeneity by detecting molecular sub‐types in NSCLC patients [27, 28].

The ‘gold standard’ source for liquid biopsy testing on *EGFR* is represented by cfDNA, recovered from plasma with several approaches [14, 29, 30]. In the present study, we showed results also obtained by using EVs as a source of RNA to identify *EGFR* mutations (codons shown in Table S4). Using the same specimens and technologies for unbiased comparison (Figs 2 and 3), the mutational profile described by the EV‐RNA qualitatively covers the one generated from cfDNA, but it reaches higher sensitivity, including a more intricate combination of simultaneous *EGFR* mutations mostly undetected in tissues and matching cfDNA. These results suggest that EV‐RNA and cfDNA work as independent biological sources in liquid biopsy in NSCLC and could provide a complementary informative set of tumor dynamics. Virtually, cfDNA is predominantly released by necrotic and apoptotic cells, while EVs are massively secreted to disseminate information to other cells and provide a variegated cargo exploitable for molecular analysis. The molecular dynamics behind this biological background could support the observation that EV‐RNA showed a reduced number of cases positive to E746_A750del (−19%) and L858R (−26%) at disease progression compared to C3D1, as indicated in Fig. 2D.

The rapid NBI‐ddPCR approach displayed, at baseline, a concordance of 92% and 89%, respectively, of E746_A750del and L858R mutations present in tumor tissue, despite 73% and 75% reported from cfDNA. Unexpectedly, the T790M appeared with high frequency (78% of cases) in EV‐RNA from baseline specimens, suggesting tumor cell sub‐clones' existence with a poorly elucidated secretome detectable by EV transcripts. So far, despite a low frequency of T790M mutation in tumor tissue at baseline, which can virtually increase using ultrasensitive methods [31, 32], other recent studies involving EVs have reported a higher frequency of T790M mutation at baseline [18, 33, 34, 35]. However, such an under‐represented mutation's clinical significance is unclear, although it is conceivable that a selection of that clone occurs during the treatment with first‐ or second‐generation TKIs, leading to an early onset of resistance. In this context, the application of quantitative approaches, as we described as a copy number of mutated transcripts in a defined number of EVs, could provide an additional parameter for assessing the clinical relevance.

Notwithstanding we did not find a significant correlation between the baseline presence of T790M mutation in EVs and tumor progression in our cohort, we report an apparent longitudinal T790M copy number increase instead of the opposite trend observed for the other two mutations—in line with previously published studies [36]—possibly suggesting a biological response/treatment pressure detectable through the vesicular RNA. Therefore, the identification of such mutation at baseline in EV‐RNA could orient the clinician to treat patients with a third‐generation TKI, such as Osimertinib, effective despite the presence of *EGFR* T790M. In the context of evaluating supporting decision tools, EVs provide material for simultaneous multidimensional analyses which could help identify tumor‐derived EV sub‐populations to enrich clinically relevant information. In our trial, the selection of EGFR‐positive EVs surprisingly resulted to an increased sensitivity for the T790M, possibly suggesting the existence of co‐segregating events from the same clonal cells.

The fact that the relative abundance of EVs released in the blood of NSCLC patients increased drastically when the disease progressed from the C3D1, with co‐occurrence of T790M mutations, indicates that vesicles can be a straightforward source to monitor the development of resistant clones upon prolonged TKI treatment. Another relevant, emerging point could rely on the relative number of EVs after the initiation of the therapy, since patients showing more EVs in circulation might also be better evaluated in response to TKIs.

## 5 Conclusions

In conclusion, this study indicated that vesicular RNA contributes a consistent picture of tumor heterogeneity and the relative abundance of *EGFR* mutations in NSCLC patients receiving TKIs. This study proposes an easily applicable protocol to integrate DNA‐ and RNA‐based parameters in longitudinal monitoring to investigate the dynamics of common driver *EGFR* mutations and improve clinical decision making.

## Conflict of interest

We thank the Fondazione Caritro, to VGD, for supporting this research. The authors declare that no conflict of interest exists, except for MN and VGD for a patent application (P019950W0) on the NBI method.

## Author contributions

LP and MN coordinated the experimental work, generated the data, analyzed the data, and prepared the original draft; AV contributed with statistical analysis; MAB collected patient data and provided clinical information; AD and LC provided oversight and mentorship external to the core team and revised the paper; EC performed sample processing, mutational analysis, and data collection; AIP provided the radiological data; PU conceived and supervised the study, reviewed, and edited the manuscript; VGD conceived and designed the experiments and supervised the study, reviewed, and edited the manuscript. All authors participated in the commentary and revision of the final work.

## Supporting information

**Fig. S1**. Specificity and sensitivity of the EV‐NBI ddPCR for 19Del, L858R, and T790M mutations.

**Fig. S2**. Characterization of EVs isolated from plasma of healthy donors and detection of *EGFR* WT in healthy and NSCLC subjects.

**Fig. S3**. Particle analysis and detection of *EGFR* mutations in EV‐RNA from *EGFR* WT NSCLC patients.

**Table S1**. Patient clinical features and follow‐up.

**Table S2**. Patient EGFR mutation analysis and EV data.

**Table S3**. EGFR EV alpha count data.

**Table S4**. EGFR primers.

Supplementary Materials

## Data Availability

The data that support the findings of this study are available in the Figs S1–S3 and Tables S1–S4 of this article.
