# Supplementary figures and images for: Unveiling mutational dynamics in non‐small cell lung cancer patients by quantitative EGFR profiling in vesicular RNA
Source: Mol Oncol. 2021 May 20;15(9):2423–38. doi: 10.1002/1878-0261.12976 (PMC8410558; doi:10.1002/1878-0261.12976)

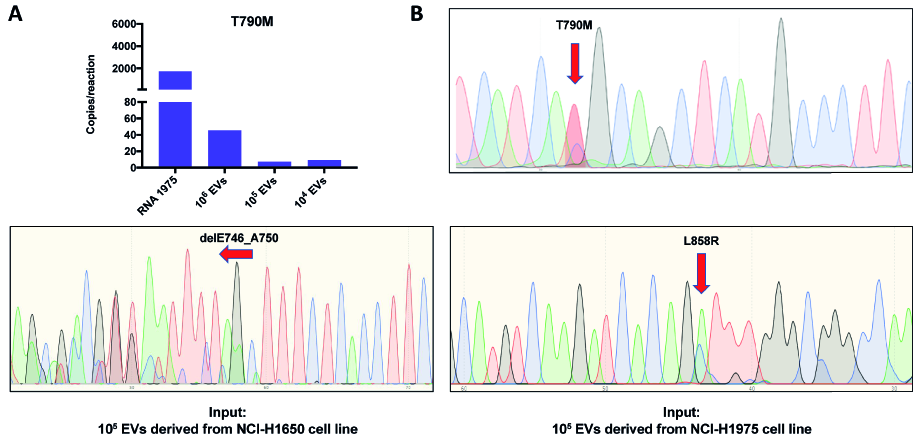

Supplement: Supplementary file 1 — Fig. S1. Specificity and sensitivity of the EV‐NBI ddPCR for 19Del, L858R, and T790M mutations. [file MOL2-15-2423-s002.tif]

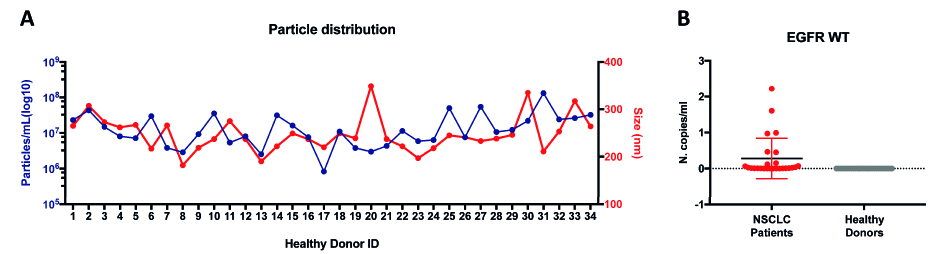

Supplement: Supplementary file 2 — Fig. S2. Characterization of EVs isolated from plasma of healthy donors and detection of EGFR WT in healthy and NSCLC subjects. [file MOL2-15-2423-s008.tif]

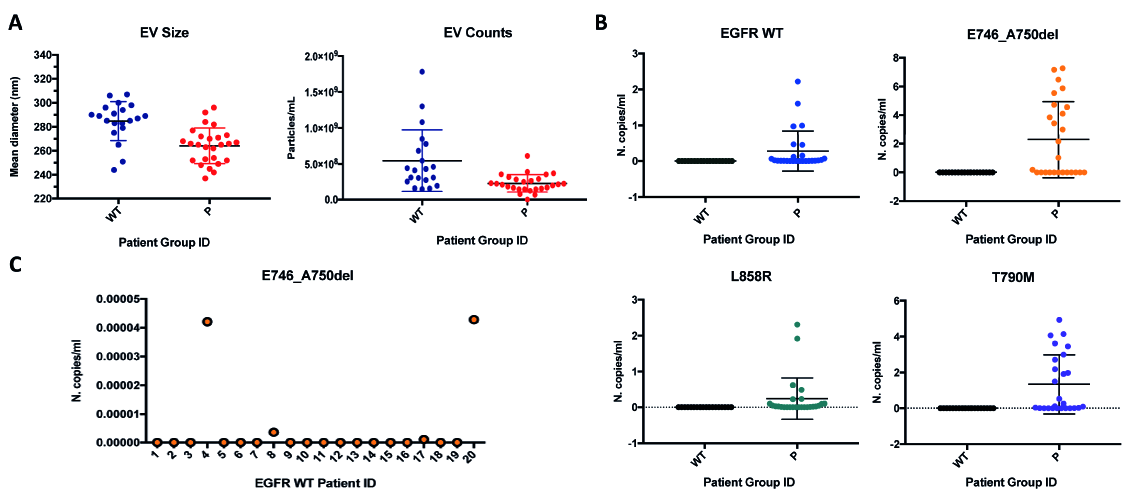

Supplement: Supplementary file 3 — Fig. S3. Particle analysis and detection of EGFR mutations in EV‐RNA from EGFR WT NSCLC patients. [file MOL2-15-2423-s001.tif]
