# Supplementary material for: Unveiling mutational dynamics in non‐small cell lung cancer patients by quantitative EGFR profiling in vesicular RNA
Source: Mol Oncol. 2021 May 20;15(9):2423–38. doi: 10.1002/1878-0261.12976 (PMC8410558; doi:10.1002/1878-0261.12976)
Supplement: Supplementary file 8 — Supplementary Materials [file MOL2-15-2423-s006.docx]

**Supplementary information**


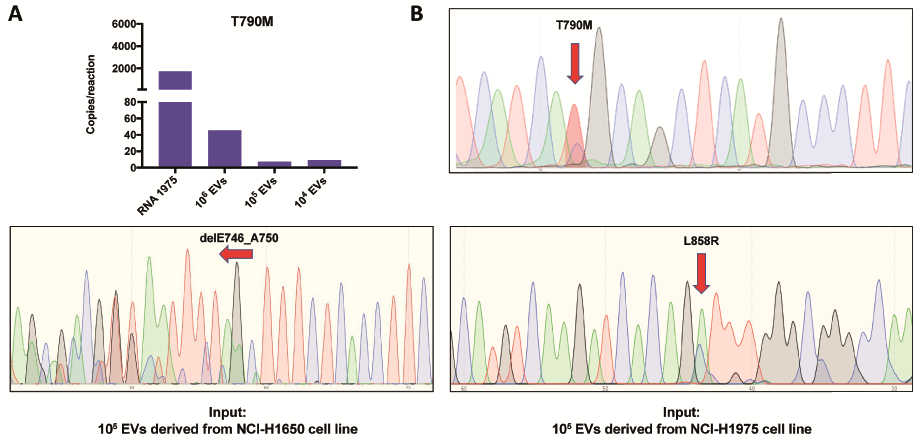


**Figure S1. Specificity and sensitivity of the EV-NBI ddPCR for 19Del, L858R, and T790M mutations. (A)** Representative plot showing the number of mutated EGFR copies (T790M) obtained by NBI-ddPCR. One nanogram of intracellular RNA was used as positive control and 10^6^, 10^5^, and 10^4^ EVs, both derived from NCI-H1975 cells, were used as templates for the ddPCR reaction. The values in the Y axis represent the number of copies per ddPCR reaction. **(B)** Partial electropherogram showing *EGFR* point mutations p.T790M and p.L858R in 10^5^ EVs isolated from NCI-H1975 cells and in-frame deletion p.E746_A750 in 10^5^ EVs isolated from NCI-H1650 cells.


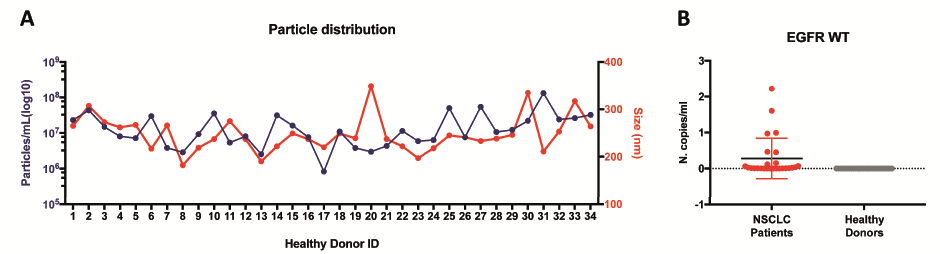


**Figure S2. Characterization of EVs isolated from plasma of healthy donors and detection of *EGFR* wild-type in healthy and NSCLC subjects. (A)** The plot shows the particle distribution profile of EVs isolated from 34 healthy volunteers screened for the presence of resistance mutation T790M and EGFR expression. Plasma from healthy donors was collected at Meyer Children’s University Hospital. Whole blood was collected into commercially available anticoagulant-treated tubes EDTA-treated (lavender tops). Informed consent was obtained from donors before the sample analysis. Particles have been analyzed by qNANO instrument with a NP250 nanopore (Izon Science). The Y-axis on the left shows the number of particles per ml of plasma (log scale) while the Y axis on the right is showing the values related to the mean diameter (nm) of isolated EVs. (**B)** The dot plot shows the number of EV-RNA copies of EGFR-wild-type (expressed as 10^10^ copies/ml) as detected by ddPCR on EVs isolated from the plasma of 34 Healthy Donors and 27 EGFR-positive NSCLC patients at baseline (mean with SD).


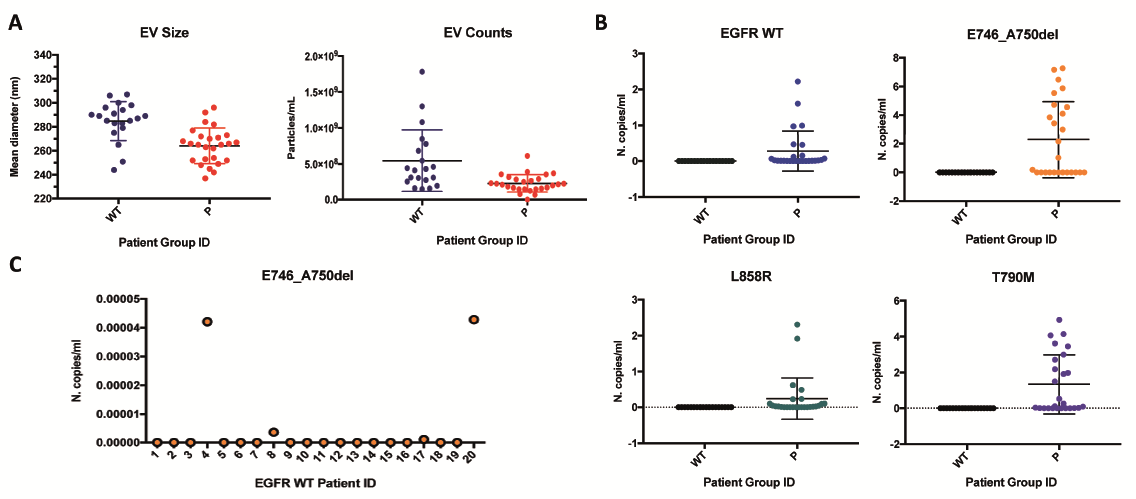


**Figure S3. Particle analysis and detection of *EGFR* mutations in EV-RNA from *EGFR* WT NSCLC patients. (A)** Plots show the analysis by Tunable Resistive Pulse Sensing (TRPS) of EVs isolated at diagnosis from NSCLC patients. On the left, the dot plot (mean with SD) represents the mean diameter (nm) of EVs isolated from 20 NSCLC patients screened as *EGFR*-wild-type (WT) in comparison with the EVs isolated from the cohort of 27 *EGFR*-positive NSCLC patients (P); on the right, the dot plot (mean with SD) shows the total number of EVs per ml of plasma of the two patient groups. (**B)** Comparisons of the number of wild-type and mutated *EGFR* copies (expressed as 10^10^ copies/ml) as detected by ddPCR on EV-RNA of EVs isolated from the NSCLC patients diagnosed with *EGFR* wild-type (WT) and the cohort of *EGFR*-positive NSCLC patients (P). (**C)** Focus on the number of mutated EV-RNA copies for the E746_A750del as detected by ddPCR in EVs isolated from the plasma of *EGFR*-wild-type NSCLC patients (expressed as 10^10^ copies/ml) at diagnosis, with two cases (#4 and #20) displaying an over-the-average amount of mutated copies of the E746_A750del.

**Supplementary tables**

**Table S1. Patient clinical features and follow-up.**

(Excel file)

**Table S2. Patient EGFR mutation analysis and EV data.**

(Excel file)

**Table S3. EGFR EV Alpha Count data.**

(Excel file)

**Table S4. EGFR Primers**

(Excel file)
